# Major dietary patterns and risk of acute myocardial infarction in young, urban Pakistani population

**DOI:** 10.12669/pjms.315.7690

**Published:** 2015

**Authors:** Romaina Iqbal, Saleem Perwaiz Iqbal, Mohsin Yakub, Asal Khan Tareen, Mohammad Perwaiz Iqbal

**Affiliations:** 1 Romaina Iqbal, PhD. Department of Community Health Sciences, Aga Khan University, Karachi, Pakistan; 2 Saleem Perwaiz Iqbal, MSc. Department of Community Health Sciences, Aga Khan University, Karachi, Pakistan; 3 Mohsin Yakub, PhD. Department of Biological and Biomedical Sciences, ***Current Address:*** Department of Biomedical Sciences, New York Institute of Technology, College of Osteopathic Medicine, Old Westbury, NY 11568, USA; 4 Asal Khan Tareen, PhD. National Institute of Cardiovascular Diseases, Karachi, Pakistan; 5 Mohammad Perwaiz Iqbal, PhD. Department of Biological and Biomedical Sciences, Aga Khan University, Karachi, Pakistan

**Keywords:** Acute myocardial infarction, dietary patterns, dietary records, Pakistani population

## Abstract

**Objective:**

To investigate the role of dietary intake in the development of premature acute myocardial infarction (AMI) in a hospital-based Pakistani population in Karachi.

**Methods:**

In a case control study, 203 consecutive patients (146 males and 57 females) with their first AMI and age below 45 years were enrolled with informed consent. Similarly, 205 gender and age matched (within 3 years) healthy adults were also included as controls. Dietary intake of both cases and controls was assessed by using a simple 14-item food frequency questionnaire. Using factor analysis, 3 major dietary patterns- prudent dietary pattern, combination dietary pattern and western dietary pattern were identified. Fasting plasma/serum of both cases and controls were analyzed for homocysteine, folate, vitamin B_12_, blood Pb, ferritin, cholesterol, LDL-cholesterol, HDL-cholesterol and triglycerides. ANOVA and conditional logistic regression were used to predict the association of dietary patterns with AMI.

**Results:**

Consumption of prudent diet, characterized by high consumption of legumes, vegetables, wheat, chicken and fruits, is protective against the risk of premature AMI. Moderate to high consumption of combination diet, characterized by high intake of eggs, fish, fruits, juices and coffee was associated with decreased risk of AMI. No association was observed between western diet, characterized by high intake of meat, fish and tea with milk and risk of AMI.

**Conclusions:**

Consumption of a prudent dietary pattern and a combination dietary pattern is protective against the risk of AMI in a Pakistani population.

## INTRODUCTION

There is growing evidence that coronary artery disease (CAD) is seen in relatively younger Pakistani population. In a published report, 28.3% of patients with acute myocardial infarction (AMI) in 17 coronary care units (CCU) in all 4 provinces of Pakistan were found to be younger than 45 years of age.^1^ In another study in Faisalabad, 35% of AMI patients admitted in CCU of Divisional Headquarter Hospital and Faisalabad Institute of Cardiology were found to be below the age of 45 years.^2^ These reports are indicative of early onset of this disease in Pakistan. An unhealthy dietary intake has been shown to increase the risk of AMI globally.^3^-^5^ Low consumption of fruits and vegetables and high intake of fat diet have been identified (among others) as risk factors for development of premature CAD.^6^ Since Pakistanis are known to consume high fat diet and low amounts of green leafy vegetables^7^-^9^, we embarked on investigating the role of dietary intake and its association with premature AMI in a hospital-based study conducted in Karachi, Pakistan.

Most researchers examine the relationship between nutrients intake or individual food items intake (such as fish intake) and their association with AMI, while food consumption is complex phenomena with most individuals consuming a mix of food items in their daily diet with both protective and harmful effects with respect to development of disease such as AMI.^10^

The study of dietary patterns has emerged as an important component of nutrition science as it allows researchers to look at the clustering of food items in diet. It also enables easy communication of health messages to the population as they are based on dietary patterns rather than nutrients or individual food items which are less meaningful. This study is a relatively large, hospital-based study conducted in Karachi, Pakistan to assess the association between dietary patterns and AMI in this population.

## METHODS

### Participants

Two hundred and three consecutive patients (146 males and 57 females) with their first AMI and below the age of 45 years admitted to the National Institute of Cardiovascular Diseases (NICVD), Karachi from June 2010 to July 2011 were included in this study with informed consent. Criteria for premature AMI were: age 18-45 years; both males and females; had confirmed diagnosis of AMI on the basis of clinical examination, ECG and biochemical data; and had no history of consuming B-vitamins (B_6_, B_12_ and folate) during the last 4 months.

Individuals who were found to be pregnant or having malabsorption syndrome or suffering from tuberculosis or liver disease, or uremia or cancer were not included in the study because these chronic diseases/conditions may lead to compromised dietary consumption and therefore could function as confounders of diet-AMI relationships. Similarly, 205 gender, and age matched (within 3 years) healthy controls from the personnel of the Aga Khan University and other health-care facilities in Karachi were also included in the study as controls. All these controls were not suffering from any of the above mentioned diseases or conditions and were not taking B vitamin supplements. Both cases and controls belonged to a low socio-economic group as 83% of controls and 90% of cases had monthly house-hold income less than US$ 150.

### Determination of food frequency

Using a simple 14-item food frequency questionnaire (FFQ), the eating habits of both cases and controls were determined. This food frequency questionnaire has been used previously in a population-based study.^11^ Information about the number of times each commonly used food item was consumed per month, per week or per day was recorded and then the frequency of each food/drink item was converted to its consumption per day as described previously.^11^ To clarify it further, if there was a response of 6 servings per month of a food item, then it was converted to 0.2 serving per day. Study had been approved by the Ethics Review Committees of the Aga Khan University as well as NICVD. Since no nutrient analysis was done, therefore a food composition table was not used.

### Measurements of biochemical parameters

Ten mL of fasting blood was obtained within 24 hours of the admission and analyzed for serum folate, homocysteine, glucose, total cholesterol, low density lipoprotein (LDL)-cholesterol, high density lipoprotein (HDL)-cholesterol, triglycerides and ferritin using commercially available kit methods (Roche Diagnostics, USA), while serum vitamin B_12_ was assayed using a radioassay.^12^ The minimum limits of detection for serum/plasma folate, homocysteine, glucose, total cholesterol, LDL-cholesterol, HDL-cholesterol, triglycerides, ferritin and vitamin B_12_ were 0.64 ng/mL, 4 mmol/L, 2 mg/dL, 9.7 mg/dL, 3.9 mg/dL, 3.0 mg/dL, 8.9 mg/dL, 0.5 ng/mL and 50 pg/mL, respectively.

### Statistical analysis

Using factor analysis, major dietary patterns were developed and then conditional logistic regression analysis was used to predict the association of dietary patterns with AMI.

Factor analysis was used to identify common dietary patterns from dietary intake data. For the generation of uncorrelated factors, factors were rotated orthogonally. Determination of number of factors to be retained in the model was carried out on the basis of Eigenvalue (>1), scree plot and factor interpretability.^13^ The analyses were run in Statistical Package for Social Sciences^®^ (SPSS; version 16 for Windows, Apache Software Foundation, USA) using the data reduction procedure. As a result, three major dietary patterns were identified in this population, which were similar to the dietary patterns identified earlier in a Pakistani population based study in Karachi.^11^ The three dietary patterns were then divided into quartiles. All cases and controls were matched for age and gender, and conditional logistic regression analysis was carried out to find out the association between each dietary pattern and AMI while adjusting for BMI, ferritin, total cholesterol, triglycerides, LDL-cholesterol and HDL-cholesterol. Values have been presented as OR (95% CI). Continuous variables have been presented as means±SD. Analysis of variance (ANOVA) was used for comparing means of quartiles across each dietary pattern. A p-value of <0.05 was considered significant.

## RESULTS

Eating habits of both cases and healthy controls were assessed using a 14-item food group frequency questionnaire (Table-I). Mean intake per day of all food items with standard deviations is reported in Table-I. Some of the food items used in this questionnaire have been previously studied for association with B-vitamins, plasma homocysteine and cardiovascular disease.^11^,^14^

**Table-I:** Factor loadings for varimax rotated factors*

Food items	Mean intake per day±SD	Prudent diet	Combination diet	Western diet
Meat	0.27±0.33	-	-	0.58
Egg	0.22±0.31	-	0.61	-
Fish	0.06±0.10	-	0.41	0.47
Chicken	0.21±0.20	0.51	-	-
Cooked vegetables	0.64±0.35	0.66	-	-
Uncooked vegetables	0.42±0.49	0.66	-	-
Legumes	0.35±0.31	0.72	-	-
Wheat	1.86±1.11	0.59	-	-
Fruits	0.46±0.40	0.37	0.53	-
Tea with milk	2.9±2.63	-	-	0.55
Tea without milk	0.14±0.56	-	-	-
Green tea	0.16±0.46	-	-	-
Coffee	0.03±0.22	-	0.34	-
Juices	0.13±0.48	-	0.49	-
Eigen value		2.46	1.32	1.18
% of variance		17.6	9.4	8.4

* Factor loadings less than 0.3 are not shown.

Factor analysis revealed 3 major dietary patterns. These were labeled as “ prudent dietary pattern”, which was characterized by high consumption of legumes, cooked and uncooked vegetables, wheat, chicken and fruits; “combination dietary pattern” which was characterized by high consumption of eggs, fish, fruits, juices and coffee; and “western dietary pattern”, which was characterized by high intake of meat, fish and tea with milk (Table-I). Each of these 3 dietary patterns was further classified into quartiles.

The descriptive information presented in Table-II shows that mean concentrations of serum ferritin and LDL-cholesterol were lower in the highest quartile of prudent dietary pattern compared to the lowest quartile (p-value <0.01). On the other hand, mean concentrations of total cholesterol, HDL-cholesterol and triglycerides were higher in the highest quartile of prudent diet compared to the lowest quartile (p-value <0.01). Regarding the western dietary pattern, individuals in the highest quartile (quartile 4) appear to have increased concentrations of serum ferritin, LDL-cholesterol and triglycerides compared to individuals in the lowest quartile, however, the values were not found to be statistically significant. It was observed that consumption of prudent diet was protective against the risk of premature AMI, however, p-value for trend was insignificant (p-value for trend > 0.05) (Table-III).

**Table-II:** Anthropometric measures and concentration of biomarkers by quartiles of dietary patterns*

Characteristics	Prudent diet			Combination diet			Western diet		
	Q^+^1	Q4	P^#^	Q^+^1	Q4	P^#^	Q^+^1	Q4	P^#^
BMI (kg/m^2^)	25.2±3.67	25.8±4.68	0.11	25.9±4.7	25.8±4.3	0.95	25.6±4.6	25.7±3.9	0.78
Homocysteine (µmol/L)	19.96±8.49	26.1±23.8	0.059	24.9±20.7	24.6±19.4	0.32	23.9±18.7	23.5±19.4	0.74
Folate (ng/mL)	6.35±3.4	7.3±3.8	0.25	6.88±3.55	6.53±3.57	0.31	6.59±3.7	6.83±3.6	0.71
Vitamin B12 (pg/mL)	318±162	362±197	0.39	335±201	350±193	0.75	344±202	340±169	0.68
Blood Pb (µg/dL)	15.2±6.6	16.3±8.49	0.096	15.57±8.7	15.29±7.1	0.53	15.81±8.6	14.22±6.3	0.34
Ferritin (ng/mL)	157±140	106±96	0.004	125±103	135±141	0.51	115±93	144±149	0.17
Cholesterol (mg/dL)	153±39	160±40	0.003	160±42	162±39	0.70	160±43	162±41	0.84
LDL-cholesterol (mg/dL)	102±34	95±35	0.006	99±40	103±33	0.79	96±37	101±33	0.155
HDL-cholesterol (mg/dL)	29.4±10.1	33.6±8.9	<0.001	33.7±13.6	33.4±10.7	0.30	34.3±10.0	31.7±11	0.38
Triglycerides (mg/dL)	107±58	156±96	0.003	140±74	142±69	0.98	136±85	152±74	0.08

* Values are means±SD

+ Lowest quartile.

# P was based on ANOVA comparing means of quartiles.

**Table-III:** OR for premature acute myocardial infarction by quartiles of different dietary patterns.

Dietary pattern	Q1	Q2	Q3	Q4	P for trend
*Prudent diet*				
Crude^+^	1	0.01 (0.00-0.06)**	0.02 (0.00-0.01)**	0.04 (0.01-0.17) **	> 0.05
Adjusted^#^	1	0.01 (0.00-0.07)**	0.01 (0.00-0.05)**	0.035 (0.01-0.25)**	> 0.05
*Combination diet*				
Crude^+^	1	0.95 (0.50-1.83)	0.31 (0.16-0.59)*	0.42 (0.22-0.80)**	< 0.05
Adjusted^#^	1	0.54 (0.19-1.55)	0.19 (0.07-0.54)**	0.27 (0.10-0.77)*	< 0.05
*Western diet*				
Crude^+^	1	1.08 (0.59-1.97)	2.01 (1.05-3.84)*	2.17 (1.15-4.09)*	< 0.05
Adjusted^#^	1	1.29 (0.52-3.24)	1.30 (0.46-3.62)	2.26 (0.83-6.18)	< 0.05

***Note:*** Q1 is the lowest quartile of dietary intake, while Q4 is the highest quartile.

+ Values are OR (95% CI) from conditional logistic regression.

# Values are OR (95% CI) from conditional logistic regression adjusted for BMI, ferritin, total cholesterol, triglycerides, LDL-cholesterol and HDL-cholesterol.

* P < 0.05

** P < 0.01

It was also observed that moderate to high consumption of combination diet was protective against the risk of premature AMI after adjusting for BMI, ferritin, total cholesterol, triglycerides, LDL-cholesterol and HDL-cholesterol (p-value < 0.05). Compared to the first quartile, the adjusted odds ratios were 0.19 (95% CI, 0.07-0.54) and 0.27 (95% CI, 0.10-0.77) for the third and fourth quartiles, respectively (p-value for trend < 0.05). Consumption of western diet was not found to be associated with the risk of premature AMI.

## DISCUSSION

Three major dietary patterns labeled as prudent dietary pattern, combination dietary pattern and western dietary pattern were obtained after using factor analysis of the 14 food items. Western dietary pattern was similar to the dietary pattern defined in an earlier study in Karachi,^15^ while prudent diet was similar to the patterns generated from INTERHEART, Health Professional Follow-up Study and Nurses’ Health Cohort.^3^,^14^,^16^ High loading of eggs in the combination diet in the present study is, however different from the afore-mentioned research studies because eggs have been part of the western diet in those studies. Other investigators have also reported a combination dietary pattern where a mix of food items belonging to different food groups has been observed.^17^ In a previous communication from our laboratory, an association of 3 dietary patterns with hyperhomocysteinemia has been reported.^11^ However, in the present study an association of some of these dietary patterns has been found with premature AMI in a Pakistani population.

Findings of this study are consistent with previous results that consumption of a prudent diet is protective against the development of heart disease after adjusting the model for BMI and other covariates as has been reported by in the INTERHEART study,^3^ and by Hu et al.^16^, however, no adverse association was observed between consumption of a western diet and risk of developing premature AMI after adjusting for the above mentioned covariates. In combination dietary pattern, an association with the risk of AMI was such that moderate to high consumption of a combination dietary pattern is associated with a reduced risk of AMI. This is indicative of a protective effect for the individuals in the third and fourth quartiles of consumption in this dietary pattern. A combination dietary pattern in Pakistani population is a unique finding of the present study. It is possible that moderate to high consumption of animal source food items combined with plant source food items exerts a beneficial effect; while no protective effect is observed when the intake is small.

In the present investigation, some biomarkers of myocardial infarction were associated with the prudent dietary pattern, while other biomarkers and physical measures were not significantly associated. Furthermore, no association was found between the combination dietary pattern and the western dietary pattern and any of the biomarkers and physical measures. Significantly low level of serum ferritin observed for individuals in the highest quartile of prudent dietary pattern compared to the lowest quartile of intake is consistent with the fact that dietary iron from plant sources is less bio-available compared to iron from animal sources,^18^ consequently individuals consuming more of the prudent dietary pattern were likely to have low serum ferritin levels. Lower mean level of LDL-cholesterol and a higher mean level of HDL-cholesterol in individuals in the highest quartile of prudent dietary intake compared to those in the lowest quartile of intake was an important observation in the current study. This is consistent with findings of a study in Korea in which individuals in the lowest quartile of a similar dietary pattern that they termed as “Korean Healthy pattern” had higher concentrations of total cholesterol and triglycerides compared to individuals in the highest quartile of intake.^19^ Relatively high serum ferritin levels observed in the highest quartile of western dietary intake compared to the lowest quartile of intake (though not statistically significant due to large variance) are suggestive that consumption of red meat could be one of the major contributors of iron to body stores.^20^,^21^ High body iron status has been reported to be associated with premature AMI in Pakistani population.^22^ Therefore, it is conjectured that increased consumption of western diet rich in red meat could be contributing to high body iron stores, thereby increasing the risk of AMI. However, results of the present investigation did not show an association of western dietary pattern with the risk of AMI in this population.

Results obtained in this investigation must be viewed within the context of certain limitations of this study. This was a case control study and there might be a possibility of recall bias between the cases and controls for dietary intake assessment. Moreover, it is likely that the cases may have changed their diets due to other preceding conditions such as hypertension or abnormal lipid profile which would minimize the association between AMI and diet. However, such a situation would lead to attenuation in the association between dietary patterns and AMI suggesting that our results might be conservative estimates. The FFQ that was used for collection of dietary data has not been validated, however similar FFQs have been used in other studies in Pakistan and they appear to have face validity.^11^ Furthermore, as a short (14 items) food intake questionnaire was used, it was not possible to estimate and consequently adjust our analysis for total energy intake. However, adjustment was made in the analysis for significant determinants of energy intake i.e. age, sex and BMI^23^, and this should lend credence to our findings. Matching of cases and controls for age and sex in the design of the study has been the main strength of this study.

This was a hospital-based study conducted at a large cardiovascular diseases hospital but larger, community based studies that would include participants from urban as well as rural areas of Pakistan are required for further confirmation of our findings.

## CONCLUSIONS

Consumption of prudent diet which is rich in legumes, vegetables, wheat, chicken and fruits is protective against the risk of premature AMI in a Pakistani population. Moderate to high intake of combination diet which is rich in eggs, fish, fruits, juices and coffee is associated with reduced risk of AMI. Western dietary pattern which is characterized by high intake of meat, fish and tea with milk does not appear to be associated with risk of AMI in Pakistani population.
